# Telehealth and Home Health Occupational Therapy: Clients' Perceived Satisfaction with and Perception of Occupational Performance

**DOI:** 10.5195/ijt.2020.6327

**Published:** 2020-12-08

**Authors:** Missi A. Zahoransky, Jennifer E. Lape

**Affiliations:** 1 Total Rehabilitation Specialists, Cleveland, Ohio, USA; 2 Chatham University, Pittsburgh, Pennsylvania, USA

**Keywords:** Canadian Occupational Performance Measure (COPM), Cost-based home telehealth, Home health care, Outcome and Assessment Information Set (OASIS), Occupational therapy, Telehealth, Telemedicine, Telerehabilitation

## Abstract

Home health care agencies are restructuring service delivery models to address quality of care and client satisfaction while containing costs. New regulatory changes and the public health emergency due to the COVID-19 pandemic precipitated an immediate need for alternative care models. Telehealth has been recognized as a feasible delivery model to provide health care. This quasi-experimental pretest-posttest study examined the feasibility of performing occupational therapy telehealth visits as an adjunct to on-site visits for homebound clients (N=9). The Outcomes and Assessment Information Set (OASIS) data collection set, Canadian Occupational Performance Measure (COPM), and a survey were used to collect data. This combination of visits resulted in clinically and statistically significant improvements in client perception of performance and satisfaction with activities of daily living. Findings showed that participants favorably perceived this service delivery model met their therapy needs and they would recommend it to others. Results of this study warrant a larger study involving physical and speech therapy services.

By 2034, for the first time in history, older adults will outnumber children in the United States (US Census Bureau, 2018). With the aging population choosing to remain in their home environment, Medicare expenditures for home health care services has increased. The Medicare Payment Advisory Commission reported that Medicare spending was $17.7 billion for home health care in fiscal year 2017 and that home health utilization increased 60% from 2002 to 2016 (MedPAC, 2019).

The public health emergency (PHE) resulting from the COVID-19 pandemic served as an impetus for all areas of healthcare to explore alternative options for care delivery. For the first time, occupational therapy practitioners could use telehealth to provide therapy services to Medicare beneficiaries as a result of expanded reimbursement through the Coronavirus Aid, Relief, and Economic Security Act (CARES Act) (AOTA, 2020). Though physicians and nurses have been utilizing telehealth for many years, telehealth adoption by other health care professionals has been relatively slow due to limited reimbursement (CDC, 2020). The COVID-19 pandemic facilitated the use of telehealth in the home health care setting by easing restrictions, reducing barriers, and providing reimbursement for telehealth services by providers not previously recognized as telehealth providers by CMS (CDC, 2020). Many organizations rapidly transitioned to telehealth to meet the needs of clients and decrease the spread of COVID-19. The American Occupational Therapy Association (AOTA) recognizes that telehealth can be an effective service delivery model across practice settings, including in the home health setting (AOTA, 2018).

Dorsey and Topol (2016) identified three trends that can directly apply to occupational therapy. One trend addressed cost containment and the second was the emergence of treating chronic conditions. The third trend, which is directly applicable to this study was the expansion of telehealth into the home environment. Telehealth has been successfully used in many practice settings, but due to limited reimbursement, its use as a service delivery model in home health care has been limited. Thus, there is a need for research to demonstrate the feasibility of telehealth as a service delivery model for occupational therapy services in the home health care setting. Due to growth of the older adult population, rising costs of health care, and a changing reimbursement climate, home health care agencies need to restructure care service models to address quality of care and client satisfaction while containing costs.

The Centers for Medicare and Medicaid Services (CMS) published the final rule for payment changes for home health care agencies and one of the primary tenets to the new payment methodology was the exclusion of any additional monies for therapy services after January 1, 2020. Under the prior payment model, therapy qualified a home health care agency for additional monies in a tier-based system, and one could argue that therapy was a revenue source for home health care agencies. In the new model of payment, although therapy appears as an expense, payment is based in part on change in functional status in clients as measured by the Outcome and Assessment Information Set (OASIS) assessment tool. Payment based on change in functional status provides a logical indication that occupational therapy services should play a prevalent role in home health care services. The 21st Century Cures Act (2016) mandated the need for information on the current use and barriers to telehealth services and dictated that CMS address telehealth within home health care. CMS (2018) clarified the definition of “remote patient monitoring” for telehealth services and stated it is now an allowable administrative cost if the home care agency uses it to “augment the care planning process.” CMS (2018) further stated that while currently there is no payment for home health telehealth services, they plan to monitor and analyze cost, impact, and client outcomes with telehealth services as well as to “consider ways to more broadly support such technology as part of home health.” CMS expressed the belief that “therapists involved in care planning, as well as other skilled professionals acting within their scope of practice, may utilize remote client monitoring to augment this process” (p. 56526). This mandate allows for telehealth visits to be part of a viable service delivery model for home health care agencies. The new payment system has agencies assessing ways to manage costs efficiently for all disciplines, with focus on overall visit numbers and determining the priority of service utilization.

It is important for occupational therapy practitioners to be as efficient as possible with limited therapy sessions as driven by payment for therapy services. As a result, it is crucial that agencies explore how alternative service delivery models may complement existing models to facilitate effective client-centered care.

The purpose of this study was to examine the effectiveness of a combination of occupational therapy on-site visits and telehealth visits (i.e., a hybrid service delivery model) on quality outcomes and client satisfaction. There have been few studies to date specifically exploring the efficacy of this model in the home health care setting.

## METHODS

A quasi-experimental pretest-posttest study was conducted by an occupational therapist (first author). The pilot study was conducted over an eight-week period in Greater Cleveland, Ohio. Participants received individualized occupational therapy home health intervention via a combination of on-site and telehealth visits. Two outcome measures, the Canadian Occupational Performance Measure (COPM) and OASIS, were administered before and after the course of intervention to assess client satisfaction and actual functional performance. At discharge, the OASIS was completed by the last discipline on the home care case. If completed by another discipline, the occupational therapist (first author) provided recommendations to complete the OASIS GG-codes, meant to measure functional changes in self-care and mobility.

An author-designed post-intervention survey was also used to measure the participants' overall perceptions of the telehealth experience including technology and use of both on-site and telehealth visits to address participant home care occupational therapy goals. The survey was divided into three parts. Part One contained five questions using a Likert scale that surveyed participants' satisfaction with the technology experience. Higher scores indicated greater satisfaction. Questions asked about client satisfaction were specific to overall device use, voice quality, visual quality, ease of use, and convenience. Part Two gathered information about participants' overall perception of the telehealth experience. Part Three collected demographic information and asked if the participants had received occupational therapy previously and if they felt occupational therapy services provided through telehealth would benefit others. The survey included two open-ended questions asking for advantages and disadvantages with using telehealth visits in conjunction with the on-site occupational therapy visits.

Inclusion and exclusion criteria were established by the first author and education was provided for all clinical staff performing Start of Care OASIS. The registered nurse or physical therapist establishing eligibility for services assessed the potential candidates for the study and completed an inclusion/exclusion criteria checklist. Inclusion criteria were: receiving homebound home health care services; 18 years of age or older; ability to see and hear; good to adequate fine motor dexterity to operate electronic device; could make own decisions about medical care; comprehended basic directions with cognitive skills permitting use of telehealth technology; ability to independently schedule appointments and tell time; a need for occupational therapy services; and agreed to receive a combination of on-site and virtual occupational therapy visits. Exclusion criteria were: a diagnosis of dementia or moderate to severe cognitive deficits that would impair ability to provide informed consent; inability to access the telehealth technology; non-English speaking; or severe low vision.

After a comprehensive occupational therapy evaluation was completed, the assignment of on-site visits and telehealth visits and the duration and frequency of visits varied by patient based on individual need. The determination for the breakdown of on-site and virtual visits was determined by the first author. A guide for service delivery model designed by the first author served as a benchmark in establishing the care plan. Clinical reasoning, clinical judgement, client needs, cultural context, professional standards of care and the AOTA Code of Ethics (AOTA, 2015) served as guidance in both the service guide delivery model development and the overall care plan decision-making process. The Telehealth Position Paper from the American Occupational Therapy Association served as additional guidance for the first author (AOTA, 2018).

On-site visits addressed areas of bathing, dressing, toileting, functional transfer training, homemaking tasks, and other privacy-sensitive tasks. Privacy-sensitive tasks involved exposure of the body. Telehealth interventions included safety education, energy conservation education, chronic care instruction, pain and medication management, activities of daily living that did not expose the body, therapeutic exercise, and review of any prior instruction provided on-site or virtually. Durable medical equipment and adaptive equipment needs with instruction were provided throughout both visit types. See the service delivery guide for this study in Appendix A. IRB approval was obtained from Chatham University.

### PARTICIPANTS

Participants were recruited from two Medicare-certified home health care agencies in the Greater Cleveland area via convenience sampling. Home health care agency nurses and physical therapists were educated in the recruitment process. A script was read, and potential participants were assessed for appropriateness to participate in the telehealth study if inclusion criteria were met. A consent form was provided to potential study participants and the first author was assigned the occupational therapy evaluation.

Participants were included in the study regardless of payor type, and initially 10 clients provided informed consent. However, one participant was admitted to the hospital after the occupational therapy evaluation and did not return home within the study timeframe; therefore, the final sample size was nine. Participants presented with a variety of primary diagnoses ranging from cardiac (n=2), orthopedic (n=3), falls (n=1), and other medical condition (n=3). Participants included eight females and one male and ranged in age from 61 to 90 years old. Table 1 includes additional participant demographics.

**Table 1:** Participant Demographics of Study Participants (N=9)

Participant	Age	Gender	Race	Primary Diagnosis	Education	Living Situation	# On site visits	# Tele-Health visits	Own device	Type of device/prior know-ledge
**A**	79	F	Caucasian	Diarrhea, Abdominal Pain	High School	With Someone	6	2	Yes	iPad Tablet/N
**B**	84	F	Caucasian	Septic Reactive Arthritis	High School	Alone	3	2	Yes	iPad Tablet/Y
**C**	90	F	Caucasian	Sepsis, UTI with IV	Bachelor Degree	With Someone	7	2	Yes	iPad Tablet/N
**D**	61	M	Caucasian	Coronary Artery Bypass Grafting x 4 Vessels	Masters+ Degree	With Someone	5	1	Yes	Dell Laptop/Y
**E**	77	F	Caucasian	Total Knee Replacement	High School	Alone	3	1	No	iPad Tablet/N
**F**	85	F	Caucasian	Femur Fracture with Pinning	Bachelor Degree	Alone	5	1	Yes	iPad tablet/N
**G**	84	F	Caucasian	Vertigo, Hypertension, Remote CVA	Bachelor Degree	Alone	3	1	Yes	iPad tablet; Smart Phone/Y
**H**	90	F	Caucasian	Falls, Transfusions, Unexplained Bruising	High School	With Someone	4	1	Yes	Samsung Tablet/N
**I**	74	F	Caucasian	Bilateral Total Knee Replacement	High School	With Someone	5	1	Yes	iPad tablet/N

### TECHNOLOGY

The technology platform used for this study was Bluestream Health. This platform met all HIPAA compliancy standards with: secure data management capacities, share-screen capability, documentation sharing features, and availability of technical resources to modify features within the platform and address technical concerns. The participants used a variety of technology devices that included the iPad tablet, Samsung Galaxy tablet, Dell laptop, and an iPhone smartphone as shown in Table 1. The technology devices were owned by the participant, a family member, or were issued for loan use within the study guidelines by the first author at the initial occupational therapy evaluation. Prior to engaging in the telehealth intervention all participants were instructed on the platform use and the home environment was assessed to ensure adequate bandwidth and/or internet or phone service. The first author reviewed the log-in process at the initial evaluation visit and trial practice was performed until the client was comfortable with the process.

### OUTCOME MEASURES

#### CANADIAN OCCUPATIONAL PERFORMANCE MEASURE (COPM)

The COPM is an individualized and self-reported measure of client satisfaction, importance, and perception of performance to a client-specific problem area in occupational performance (Law et al., 2014). This tool is designed to assess the client's perception of performance and supports client-centered care. The COPM was used to identify problem areas in the client's occupational performance and assisted in establishing therapy goals. Importance of performance area, perception of performance of task, and satisfaction of performance were rated by the participants on a scale of 1-10, with 10 being the higher score. Research indicates that the COPM has high content and construct validity, responsiveness to change over time, interpretability and feasibility (Tuntland et al., 2016), which made it a good fit for this study. This assessment was administered at the beginning and end of the occupational therapy course of treatment.

#### OUTCOMES AND ASSESSMENT INFORMATION SET (OASIS)

The OASIS is within the realm of public domain and is embedded in the medical record for each home health care client. The OASIS GG-codes address specific areas of activities of daily living, functional mobility and safety. Research findings on the validity and reliability of the OASIS demonstrates the tool accurately measures outcomes for home health care clients (Tullai-McGuinness et al., 2009). The OASIS provides constructive data on the impact of occupational therapy on areas of activities of daily living and instrumental activities of daily living, to validate impact of home health care services on occupational performance. This measurement tool allowed for data collection and analysis of clients' occupational performance for this study. The OASIS is completed at the start of home health care and at discharge. At the start of care the OASIS was completed by the admitting registered nurse or physical therapist; the first author (an occupational therapist) provided recommendations to the completing clinician for scoring on GG codes. The discharge OASIS was completed by the last discipline in the client's care with feedback from the care team for accurate scoring of the GG codes.

#### POST-INTERVENTION SURVEY

The first author developed a post-intervention survey with Likert-type questions and open-ended questions. After development, the survey was reviewed by experts within the fields of telehealth and occupational therapy to assess for relevance, clarity, and inclusion of needed data items. Expert feedback included recommendations to add and delete items, clarify the wording of questions, and make format changes. Any difference of opinion was discussed until consensus was achieved. Modifications to the tool were made based on the experts' feedback. The final version of the survey collected demographic information and measured participants' perception and satisfaction with a combination of on-site and virtual occupational therapy visits.

### PROCEDURES

The study was implemented in four phases over the eight-week period consisting of: initial visit and pre-intervention outcome measures, intervention, discharge visit, and post-intervention outcome measures. See Table 2 for an illustration of the steps completed for each phase of the study.

**Table 2:** Phases of Study with In-Phase Steps

Initial Visit and Pre-intervention Outcome Measures	Intervention	Discharge Visit	Post-intervention Outcome Measures
Informed Consent reviewed, questions answered, and signed consent collected Occupational therapy evaluation COPM administered and OASIS data collected Technology device determination and platform instruction	Occupational therapy on-site visits in combination with telehealth visits Review of occupational therapy plan of care Modification of service delivery guide Documentation of visit with plan established for next visit(s)	Final on-site intervention as per agency guidelines Collection of post-intervention survey if completed Loaned technology collected	Post-Intervention Survey COPM OASIS

For each participant, the occupational therapy evaluation was completed by the first author per Medicare/agency guidelines. Findings were discussed with the participant and a client-centered plan of care was developed. The first author determined the breakdown of on-site visits and virtual visits and noted them on the participant's calendar. The COPM data were collected by asking participants to identify areas they wanted to address during therapy. The participants further scored the measure as per assessment instructions and the data were recorded on the COPM form. The first author completed an OASIS coding form which factored into the participants' overall GG code scoring on the Start of Care OASIS.

The intervention visits followed the physician-signed plan of care. All telehealth intervention visits were performed by the first author. On-site visits were performed by the first author or a certified occupational therapy assistant, which is standard practice for this setting. The number of on-site visits per participant varied from three to seven visits and the telehealth visits varied from one to two visits per participant. The duration of on-site visits ranged from 45-75 minutes and the telehealth visits ranged from 23-42 minutes. The discharge visit was on-site and included administration of all study outcome measures.

## DATA ANALYSIS

Quantitative data were analyzed via descriptive and inferential statistics using the SPSS software Version 23 program. Cohen's d was manually calculated. Qualitative data collected from the post-intervention survey were transferred to Microsoft Excel Version 16 for analysis. Data were reviewed by the first author and the second author independently for investigator triangulation. The data were coded into themes individually and any disagreements were resolved through discussion until consensus was reached. Inductive analysis was applied, and commonalities were identified.

## RESULTS

### QUANTITATIVE RESULTS

#### COPM

***Importance.*** Participants were asked to identify up to five occupational performance problems they wanted to address. This assessment tool was used to measure each participant's perception of occupational performance and satisfaction from start to completion of occupational therapy intervention. Each participant was asked to rate the importance of each identified occupational performance problem on a scale of 1-10 with 10 being most important. Participants were allowed the opportunity to list more than five problems but then the participant and first author ranked the top five by level of importance. Participant importance rankings varied with one participant scoring a three and a four on two identified problems, but most scores were from nine to ten with a mean of 8.86. There was no correlation between ranking of importance and amount of change from pre-score to post-score in performance or satisfaction.

**Figure 1:**
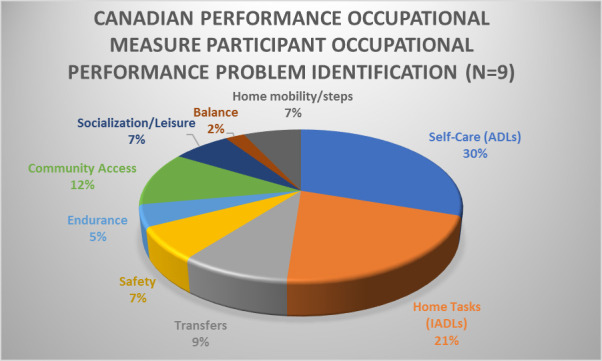
Percentage of identified performance areas

*Note:* Percentage of identified performance areas from the Canadian Occupational Performance Measure (n=9).

***Performance and satisfaction.*** A total of 43 occupational performance problems were identified. These were categorized into nine areas: self-care (30%), IADLs (21%), community access (12%), transfers (9%), home mobility/steps (7%), safety (7%), socialization/leisure (7%), endurance (5%), and balance (2%). See Figure 1 for an illustration of identified areas of performance problems.

Each participant rated the performance of the identified occupational performance problem on a scale of 1-10 with 10 representing able to do it extremely well. The mean of the participants' pre-performance scores was +2.33 and the mean of the post-performance scores was +8.56. The mean score for change for all participants in all occupational performance problems was +6.23.

Participants rated satisfaction of the identified occupational performance problem on a scale of 1-10 with 10 representing extremely satisfied. Participant pre-satisfaction scores had a mean of +2.56 and participants' post-satisfaction scores had a mean of +8.95. The mean score for change for all participants for satisfaction was +6.4. A higher score indicates an improvement and all 43 identified occupational performance problems showed improvement in both performance and satisfaction. Table 3 compares the occupational performance problems pre- and post-scores identified by participants and identifies overall change in each area.

**Table 3:** Comparison of Pre- and Post-Canadian Occupational Performance Measure Data

Performance			Satisfaction					
Participant	Occupational Problem	Importance	Pre	Post	Change	Pre	Post	Change
**A**	Showering Dressing Living area access Meal prep/cleanup Community access	8 10 10 10 9	1 1 1 1 1	7 9 9 8 5	**6** **8** **8** **7** **4**	1 1 1 1 1	8 9 10 8 7	**7** **8** **9** **7** **6**
**B**	Toileting hygiene Transfers Endurance	9 10 10	5 7 6	10 10 9	**5** **3** **3**	5 6 5	10 10 9	**5** **4** **4**
**C**	Dressing Showering Socialization/Leisure Transfers Community Access	9 9 10 10 8	2 1 1 2 1	10 9 9 8 10	**8** **8** **8** **6** **9**	2 1 1 3 5	10 10 10 9 10	**8** **9** **9** **6** **5**
**D**	Showering Endurance Functional tasks Transfers Safety in home	7 10 10 6 5	2 1 1 5 3	8 7 9 7 9	**6** **6** **8** **2** **6**	1 1 1 2 2	9 9 9 8 9	**8** **8** **8** **6** **7**
**E**	Showering Dressing Safety Community access Laundry	10 10 10 10 8	1 3 1 3 1	8 9 7 9 9	**7** **6** **6** **6** **8**	1 3 1 1 1	7 9 7 9 10	**6** **6** **6** **8** **9**
**F**	Dressing Transfers Community access Socialization Home tasks	8 4 3 9 9	7 1 1 1 1	9 9 9 9 5	**2** **8** **8** **8** **4**	8 1 1 1 1	9 9 9 9 7	**1** **8** **8** **8** **6**
**G**	Showering Carrying items Balance Community access Home tasks	9 8 10 9 10	5 4 5 1 5	9 9 9 8 9	**4** **5** **4** **7** **4**	3 2 4 3 5	9 9 10 8 10	**6** **7** **6** **5** **5**
**H**	Showering/Dressing Cane for safety Cooking Laundry Helping care for daughter	10 10 10 10 9	6 2 1 2 1	10 9 9 9 7	**4** **7** **8** **7** **6**	5 3 2 3 3	10 9 9 10 8	**5** **6** **7** **7** **5**
**I**	Shower in tub Dress self Steps to upstairs Sleep in bed Cook/laundry	9 9 10 10 7	1 2 1 1 1	9 8 8 10 9	**8** **6** **7** **9** **8**	3 3 4 3 5	9 8 8 10 9	**6** **5** **4** **7** **4**
**MEAN**	**8.86**	**2.33**	**8.56**	**6.23**	**2.56**	**8.95**	**6.4**

**Figure 2:**
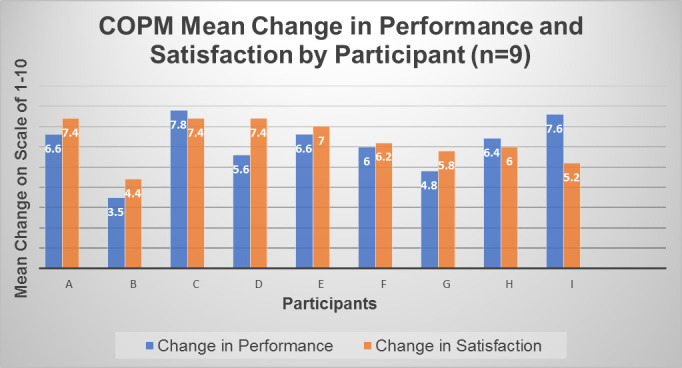
Individual Participant's Average Improvement in Perceived Performance and Satisfaction

*Note.* Comparison of individual participant's improvement in perceived performance and satisfaction.

Individual improvement averages of all identified occupational performance problems ranged from +3.5 to +7.8 points for performance and +4.4 to +7.4 points for satisfaction. For all participants there was improvement for all identified occupational performance problems. Figure 2 compares the individual participants' average improvement in perceived performance and satisfaction.

Paired sample t-test (pre-test vs. post-test) yielded a t-value of 21.65 for performance and 24.78 for satisfaction. These extremely large values were significant well beyond a p=value of <.001. The effects sizes for the COPM as indicated by Cohen's d was high. See Table 4 for statistical analysis findings for the Canadian Occupational Performance Measure paired sample test and Cohen's d.

**Table 4:** Statistical analysis for COPM

	Paired Differences									
	# Problem areas	Mean	SD	SE	t	df	Sig. (2-tailed)	p value	Significance	Cohen's d
Pre-Post Performance	43	2.33-8.56	1.88	.288	21.65	42	.000	<.001	Highly Significant	3.31
Pre-Post Satisfaction	43	2.56-8.95	1.69	.258	24.78	42	.000	<.001	Highly Significant	3.78

*Note:* Table shows statistical analysis for COPM mean, standard deviation (SD), standard error mean (SE), t-value, p-value, and significance based off paired t-test for all nine participants.

**OASIS.** The OASIS GG-codes addressed specific areas of activities of daily living, functional mobility, and safety. The GG0100 code looked at four prior functional categories of self-care, ambulation, stairs, and functional cognition. Eight of nine participants were independent in self-care prior to their current illness, injury, or exacerbation. Seven were independent with ambulation with two participants requiring some assistance prior to admission. Previously, five participants were independent with stairs, two required some assistance, and two had no stairs in their living environment. All participants were scored as independent for functional cognition prior to their current illness, injury, or exacerbation as well as during the initial occupational therapy visit. GG0110 captured each participant's prior mobility device use. Four of the participants had no prior device use and five had prior device use.

**Figure 3:**
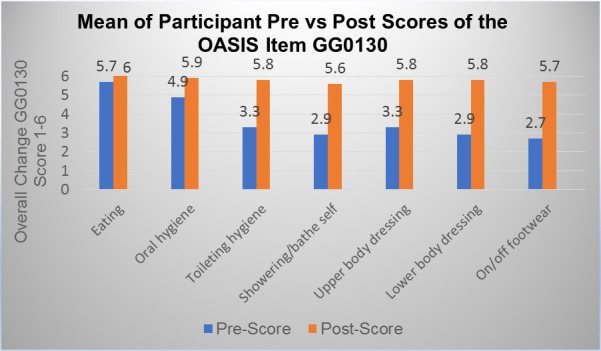
OASIS GG0130

*Note.* OASIS GG0130 overall participant pre- and post-change scores in eating, oral hygiene, toileting hygiene, showering/bathing, upper and lower body dressing, and putting on/taking off footwear. A higher score indicates a higher level of function.

GG0130 measured each participant's self-care safety and quality of performance at start of care and at discharge. Self-care tasks measured were eating, oral hygiene, toileting hygiene, showering/bathing, upper and lower body dressing, and putting on/taking off footwear. Scores ranged from 1-Dependent to 6-Independent with an overall pre-score mean of 3.67 and post-score mean of 5.78. Figure 3 illustrates overall participant pre- and post-score change in self-care measures.

GG0170 captures a participant's performance in mobility for 20 measures. Scored measures of mobility included bed mobility (rolling, lying to sitting, and sit to lying); transfers (sit to stand, bed/chair, toilet, and car); walking (10 feet, 50 feet, 150 feet, and 10 feet uneven surfaces); steps (1 step, 4 steps, and 12 steps) and picking up an object. The final five measures address wheelchair use and ability (e.g., propelling and navigating wheelchair). In this study one participant used a wheelchair prior to home care and continued this use after discharge. The scoring criteria is the same as for GG0130. Scoring for all measures occurred at start of care and at discharge.

Paired sample t-test comparing pre- and post-test ratings showed a *t-*value of 12.80 for GG0130 (*p-*value of Ã.001) and a value of 15.39 (*p*-value Ã.001) for GG0170. The effects sizes for OASIS as indicated by Cohen's d was high. See Table 5 for statistical analysis findings for the OASIS GG0130 and GG0170 paired sample test and Cohen's d.

**Table 5:** OASIS Comparison

	Paired Differences									
	# areas	Mean	SD	SE	t	df	Sig. (2-tailed)	p value	Significance	Cohen's d
Pre-Post GG0130	63	3.67-5.78	1.31	.165	12.80	62	.000	<.001	Highly Significant	1.61
Pre-Post GG0170	104	3.24-5.28	1.35	.132	15.39	103	.000	<.001	Highly Significant	1.51

*Note.* Shows statistical analysis for GG0130 and GG0170 for mean, standard deviation (SD), standard error mean (SE), t-value, p-value, and significance based off paired t-test for all nine participants.

#### POST-INTERVENTION SURVEY

All but one of the participants answered ‘Satisfied' or ‘Very Satisfied' in the categories of technology use, voice quality, visual quality, and convenience. One participant answered ‘Dissatisfied' with technology use and visual quality, ‘Very Dissatisfied' with ease of use of device and ‘No Opinion' for convenience. Eight of the nine participants felt the combination of telehealth visits with on-site visits met their needs and if they needed occupational therapy in the future, they would be willing to receive intervention with the combination of both types of visits. Six of the participants had received previous occupational therapy and three had never received occupational therapy prior to this home care admission. It should be noted that this question was asking about any occupational therapy intervention such as hospital or skilled nursing facility as well as home care. Eight of the nine participants responded that they felt others could benefit from occupational therapy services delivered through telehealth. The post-intervention survey data is illustrated in Appendix B.

### QUALITATIVE RESULTS

#### POST-INTERVENTION SURVEY

Participant responses indicated three predominant themes in relation to advantages for the combination of in-person and telehealth visits. The first theme identified was increased opportunity for both the participant and the clinician. This theme was inclusive of participant reported statements about “opportunity for further instruction” and “opportunity for real time instruction.” The second theme was convenience, and one participant noted how it was “…easier for the therapist. There is no travel time or bad weather to contend with” while another stated “Discussion was not impeded [sic] and time and travel saving was significant compared to a ‘traditional' visit.” The final theme of quicker response time was supported by statements of “Can react quick to an unplanned opportunity” and “It's handy and can handle a small problem right away.” For disadvantages two themes emerged: preferring on-site visits and technology challenges. See Appendix B for sample quotes from the participants specific to identified themes.

Additional participants' responses provided information to yes/no questions asking if the participants felt the combination of visits met their needs and if they would receive occupational therapy services again with both on-site and telehealth visits. In regard to feeling the combination of visits met their needs and if participants would recommend this approach to care, participants stated: “I also enjoyed the discussion on my progress with (first author) especially when she noticed slight changes in my posture, expression” and “I think you hit the most important points quickly with this dual approach.” For those who answered ‘no,' statements included: “Not enough exposure to know whether I would appreciate using it” and “It was nerve wracking. I am afraid I will be expected to receive instruction for sx [sic] over my phone. No thank you.” The last question allowed participants to include any additional comments they wanted to share. Participants shared overall statements such as “Having OT got me back to where I was before my illness” and “It was a very positive experience.” Participants' explanatory quotes can be seen in Appendix B.

## DISCUSSION

Because of expansive home health reimbursement changes and reductions, there is a need to explore alternative service delivery models for therapy services that demonstrate improved outcomes and client satisfaction. One caveat for exploring alternate service delivery models is to assure that client-centered care is not negatively impacted. Client-centered care is assessed by examining client perception of satisfaction and clinical measures of performance improvement.

The purpose of this study was to determine if a combination of on-site home health occupational therapy visits and telehealth occupational therapy visits would improve the homebound clients' perceived satisfaction with and perception of occupational performance. The findings of the study support use of this combination of visits and suggest this may be a viable alternative service delivery model for providing occupational therapy interventions in the home care setting. Furthermore, the findings support that the client-centered care model is not negatively impacted with the use of telehealth, but in fact, telehealth is perceived positively by clients.

### TELEHEALTH

This study focused on the use of telehealth and did not include pre-determined parameters on diagnosis or age of participants. While a study by Nelson et al. (2017) demonstrated that older adults might be less likely to want to initially participate in telehealth, the findings in this study indicated that age did not factor into willingness or success of the telehealth intervention portion of the care. Six of the participants did not have any prior experience with the technology; this did not impact the overall results of improvement in all areas of performance measured. While much of the evidence surrounding telehealth use addresses clients with specific diagnoses (Boehm et al., 2015; Dunleavy et al., 2013; Fitzsimmons et al., 2016; Gorst et al., 2016; Hwang et al., 2017; Marquis et al., 2014; Nelson et al., 2017; Radhakrishnan et al., 2016; Renda & Lape, 2018; Tousignant et al., 2014; Yuen et al., 2015), this study had no restrictions in place related to diagnosis and provides both preliminary support and new evidence to suggest telehealth may be appropriate for a variety of diagnoses in the traditional home care setting.

Some participants required more involved instruction initially on how to use the technology but none of the findings indicated that prior knowledge of technology, age, diagnosis, or caregiver supports played a role in their overall use of telehealth for occupational therapy intervention. The findings indicated that most participants were either ‘Very satisfied' or ‘Satisfied' with the measured areas of technology use. See Appendix B for illustrated results.

The findings of this study support prior research that suggests telehealth is a viable option the delivery of therapy services in a community-based model of care (Boehm et al., 2015; Fitzsimmons et al., 2016; Gorst et al., 2016; Grant et al., 2015; Hwang et al., 2017; Levy et al., 2015; Marquis et al., 2014; Nelson et al., 2017; Renda & Lape, 2018; Tousignant et al., 2014). This study specifically demonstrates that telehealth can be a viable option for the homebound home care client. The findings support that telehealth can be an effective service delivery model when virtual visits are provided in conjunction with on-site visits with all but one of the participants reporting satisfaction with this model. This participant did not feel the combination of visits met their needs, nor did they recommend this treatment model for others. They cited anxiety over technology use and concern that “I do not want this technology to take anyones [sic] job.” In prior studies, clients felt telehealth was an option but preferred in-person visits; however, overall changes in client satisfaction and perception scores were not statistically significant in studies of either onsite-site or telehealth visits (Boehn et al., 2015; Fitzsimmons et al., 2016; Gorst et al., 2016). While qualitative findings from this study supported the preference for on-site visits, statistically significant improvements for both client satisfaction and perceptions of improvement were noted with the use of a combination of onsite and telehealth visits. This may indicate increased comfort with technology when the opportunity also exists for in-person interaction.

### OUTCOME MEASURES

Both the COPM and the post-intervention survey were client self-reported measures. OASIS is a clinically driven assessment tool. Both types of outcome measures were important to explore as the government publishes publicly reported outcomes on both performance outcomes and client satisfaction for viewing by the public, as well as referral sources. A home care agency's survival can be impacted by this publicly available data. The findings demonstrated that with the use of on-site and telehealth visits, participants' demonstrated improvements in all 43 identified problem areas on the COPM. Much of the literature supports client reported improvements in *either* satisfaction or functional performance improvement (Grant et al., 2015; Hwang et al., 2017; Levy et al., 2015). This study found that by utilizing the use of a combination of on-site and telehealth visits, all nine participants demonstrated highly statistically significant improvements in both performance and satisfaction post occupational therapy intervention. A change of two points on the COPM measure is seen as clinically significant. With a mean change score for all participants in both performance and satisfaction greater than six points, the findings support that telehealth visits in conjunction with on-site visits is both a clinically and statistically significant alternative service delivery model.

Based on a client-centered approach, each participant identified a different list of problems. Review of the literature identified functional mobility as a highly identified problem (Donnelly et al., 2017; Renda & Lape, 2018). Findings for this study indicated that self-care, specifically showering, plays an important role in the rehabilitation needs of the homebound client and was identified as the top priority in six of the nine participants. This study began to identify what interventions would be feasible for on-site and telehealth visits and correlated these interventions to identified practice patterns in addressing goals. For example, a shower was identified as an on-site visit but the discussion on DME and adaptive equipment needs was accomplished successfully within the virtual visit.

The quantitative findings indicated that participants demonstrated statistically and clinically significant improvements in all areas of client perception and clinician assessed performance outcomes. The qualitative findings indicated that participants felt the combination of in-person and telehealth visits provided a good opportunity, quicker response, and convenience. The study results also indicated that while participants might prefer on-site visits, participants felt that the combination of on-site and telehealth visits met their needs, they would receive occupational therapy services again in this manner, and they would recommend this service delivery model to other home care clients.

The clinically measured OASIS GG0130 and GG0170 indicated that for areas of self-care and functional mobility the combination of on-site and telehealth visits was a viable service delivery model. All participants' demonstrated highly statistically significant improvements in both GG0130 (self-care) and GG0170 (functional mobility) post occupational therapy intervention. This study used three outcome measures to collect data. Results indicate that the participant perceived improvements in performance and satisfaction with performance (COPM), and the clinically assessed participant improvement (OASIS) were statistically and clinically significant.

### LIMITATIONS

The small homogenous sample size from one geographic area decreases the generalizability of the findings to a larger population.

Another limitation was that all participants were found after intervention to have a high school degree or higher. This could have impacted the ability to engage in the study and follow the technology directions.

Furthermore, one anticipated issue in the use of technology for telehealth services is cost. While this study did not find any insurmountable challenges specific to technology, the sample size and timeframe were too limiting to explore costs.

The nature of the outcome measures may also be a limitation. The COPM is a self-report measure and the OASIS is a clinician reporting measurement tool that could have allowed for participant or researcher bias.

The timeframe of the study was eight weeks and did not allow for long-term follow-up. This lack of follow-up limits the ability to understand and analyze the long-term outcomes. The timeframe also limits the ability to address sustainability and identify any additional barriers to the use of telehealth in the home health care setting that may occur.

### IMPLICATIONS

This pilot study adds to the body of knowledge for feasibility of telehealth utilization in providing occupational therapy visits in home care with a combination of both on-site and telehealth visits. This study demonstrated positive client perceptions of satisfaction and occupational performance improvement at a highly significant level. The application of this study to the homebound client adds evidence to a changing area of practice for the home care occupational therapist. Telehealth has been identified as a future service delivery model in home care (CMS, 2018) as well as supported as an appropriate service delivery model for occupational therapy practitioners (AOTA, 2018; Cason, 2015). This pilot study's findings support initiatives to expand the use of telehealth as a viable service delivery model for occupational therapy in traditional home care. There is a need for further research to evaluate the efficacy of home health care services provided exclusively through telehealth and through a hybrid approach, wherein some services are provided in-person and others through telehealth (Levy et al., 2015; Nelson et al., 2017; Nobakht et al., 2017). To fully assess telehealth in a client-centered model of care both quantitative and qualitative factors must be considered.

Expanding the study question to include all three therapy disciplines (occupational therapy, physical therapy, and speech therapy) would provide an interdisciplinary approach that could allow professionals to advocate for maintaining reimbursement for services provided through telehealth, especially after the COVID-19 public health emergency has ended. A longitudinal study would be warranted to explore developmental trends and improve efficacy of determining variable practice patterns over time. Similar studies and additional research are needed to more extensively address the correlation of the clinical component and the client-driven component of occupational performance improvement. Further research studies to address clinical implications of telehealth use in home care such as clinical skill sets necessary, service delivery guides, and exploration of cost implications are needed. Exploration of comparative data utilizing the OASIS outcome measure for performance improvements from all on-site visits and a combination of on-site and telehealth visits is planned as a follow-up study.

## CONCLUSION

As healthcare policy and reimbursement restructuring continues, these changes will continue to challenge the home health care system. The global coronavirus pandemic has further catapulted telehealth into a national narrative and studies such as this provide evidence that support alternative client-centered service delivery models while maintaining quality outcomes and patient satisfaction. The findings from this study add to the much-needed evidence to support telehealth initiatives and future projections for the provision of home health care services. This pilot study could serve to support future policy initiatives related to the provision of therapy services through telehealth. Finally, this study suggests the use of telehealth for the traditional home care population with a combination of on-site and virtual visits may serve as a viable service delivery model for home care agencies and home care clients.

## APPENDIX A SERVICE DELIVERY GUIDE

### SERVICE DELIVERY GUIDE

#### TELEHEALTH OCCUPATIONAL THERAPY INTERVENTION

**The following form is to establish guidelines on occupational therapy interventions that are appropriate for on-site visit and telehealth visits. This list is a guideline and is not to replace the clinical judgement of the occupational therapist. Each patient's need will vary, and an appropriate combination of on-site and telehealth visits should be established and intervention appropriately delineated within each visit type.**

**On-Site Visit:**
- Evaluation
- Activities of daily living (ADLs): The following tasks would be on-site due to nature of intervention if the patient will be naked or is of a personal manner where modesty cannot be maintained in a virtual visit:
  - Bathing
  - Dressing
  - Toileting
  - Grooming
  - Feeding
  - Functional Transfer training/functional mobility
  - Any other tasks involving safety or privacy needs
- Instrumental activities of daily living (IADLs): The following tasks would be on-site due to nature of intervention:
  - Meal preparation
  - Housekeeping tasks
  - Home maintenance tasks (mail, garbage, outside yardwork)
  - Any tasks requiring close visualization (finances, telephone use)
- Discharge visit

**Telehealth Visit:**
- Patient monitoring/teach-back of previous intervention material
- Safety education
- Process measure and chronic condition instruction
- Energy conservation
- Falls education
- Activities of daily living (ADLs): Tasks such as socks/shoes; brace don/doffing; feeding; grooming; transfers once at level of safety with/without device
- Therapeutic Exercise Programs
- Pain and medication management

## APPENDIX B

**Table:** Participants Post-Intervention Survey Quantitative Results

Question	*n*	%
**How satisfied were you using the tablet for your telehealth OT sessions?**		
Very Dissatisfied	0	0%
Dissatisfied	1	11.1%
No Opinion	0	0%
Satisfied	2	22.2%
Very Satisfied	6	66.7%
**How satisfied were you with the Voice quality of the tablet for your telehealth OT sessions?**		
Very Dissatisfied	0	0%
Dissatisfied	0	0%
No Opinion	1	11.1%
Satisfied	3	33.3%
Very Satisfied	5	55.6%
**How satisfied were you with the Visual quality of the tablet for your telehealth OT sessions?**		
Very Dissatisfied	0	0%
Dissatisfied	1	11.1%
No Opinion	0	0%
Satisfied	4	44.45%
Very Satisfied	4	44.45%
**How satisfied were you with the Ease of Use of the tablet for your telehealth OT sessions?**		
Very Dissatisfied	1	11.1%
Dissatisfied	0	0%
No Opinion	1	11.1%
Satisfied	1	11.1%
Very Satisfied	6	66.7%
**How satisfied were you with the Convenience of the tablet for your telehealth OT sessions?**		
Very Dissatisfied	0	0%
Dissatisfied	0	0%
No Opinion	1	11.1%
Satisfied	3	33.3%
Very Satisfied	5	55.6%
**Did you feel that the combination of both telehealth visits and on-site visits for occupational therapy treatment met your needs?**		
Yes	8	88.9%
No	0	0%
No response	1	11.1%
**If you needed occupational therapy treatment again in the future would you be willing to receive both telehealth and on-site visits again?**		
Yes	8	88.9%
No	1	11.1%
**Have you received home health occupational therapy services before?**		
Yes	3	33.3%
No	6	66.7%
**Do you feel that other patients could benefit from telehealth when receiving home health occupational therapy services?**		
Yes	8	88.9%
No	1	11.1%

**Table:** Participant Post-Intervention Survey Quotes and Qualitative Themes

Theme: Advantages of Telehealth	Quotes
**Opportunity**	“Opportunity for further instruction” “Opportunity to observe the environment” “Opportunity for real time instruction” “Opportunity for reinstruction” “Good experience” “I liked it”
**Convenience**	“Just being able to do the exercises on your own at the time you want to” “No advantage for me however, it is much easier for the therapist” “There is no travel time or bad weather to contend with” “Discussion was not impeded [sic] and time and travel saving were significant compared to a “traditional” visit” “Logging on was very easy and convenient for my purpose” “It was a time saver” “Good contact at anytime”
**Quicker response time**	“Can react quick to an unplanned opportunity” “Its [sic] handy and can handle a small problem right away”
**Theme: Disadvantages of Telehealth**	**Quote**
**Preferring on-site visits**	“May not be good for bathing or if you need help walking” “I do not think it should be used in place of hand [sic] on treatment. Only used 1x. Was anxious and intimidated by the whole process” “As a 90 y.o. [sic] I think that I would like a person actually showing up in person. That way I can ask questions while I am thinking about it” “It wasn't as personal as I would have liked”
**Technology challenges**	“Set up may pose obstacles in areas mostly electronic” “Had problems seeing (first author) at first-no visual”

**Table:** Participants Post-Intervention Survey Explanatory Quotes

Question	Quotes
**Did you feel that the combination of both telehealth visits and on-site visits for occupational therapy treatment met your needs?**	Response ‘*Yes':* “I also enjoyed the discussion on my progress with (first author) especially when she noticed slight changes in my posture, expression” “Consultations or strictly dialoging could occur electronically as effective as a traditional visit” “Good support mechanisms for learning and reinstruction as well as achievement” Response ‘*No':* “Not enough exposure to know whether I would appreciate using it” “Some things don't translate as well over a TV camera” “I do not want this technology to take anyone's job”
**If you needed occupational therapy treatment again in the future would you be willing to receive both telehealth and on-site visits again?**	Response ‘*Yes':* “I think you hit the most important points quickly with this dual approach” “Enhances contact for continuous learning as obstacles for such” Response *‘No’:* “It was nerve wracking. I am afraid I will be expected to receive instruction for sx [sic] over my phone. No thank you”
**Additional Comments: Please feel free to add any comments the survey did not ask or you would like to share about your experience.**	“Having OT got me back to where I was before my illness” “I believe in the importance of person-to-person meetings. Telehealth is great in time management-saving in travel and quick access to therapist/patient” “It was a very positive experience” “A super experience” “I feel I was able to meet my challenges and achieve my goals”
